# Comparing patients and families perceptions of satisfaction and predictors of overall satisfaction in the emergency department

**DOI:** 10.1371/journal.pone.0221087

**Published:** 2019-08-13

**Authors:** Prathiba Natesan, Dima Hadid, Yara Abou Harb, Eveline Hitti

**Affiliations:** 1 Department of Educational Psychology, University of North Texas, Denton, Texas, United States of America; 2 Department of Emergency Medicine, American University of Beirut, Beirut, Lebanon; 3 Patient Affairs Office, American University of Beirut, Beirut, Lebanon; University of South Australia, AUSTRALIA

## Abstract

**Study objective:**

The aim of the study was to investigate factors that best predict patient’s satisfaction with their ED visit, as well as examine whether patients and their families perceived the factors related to satisfaction similarly.

**Methods:**

This is a retrospective study where secondary data analysis was done on patient satisfaction data collected over three quarters for quality improvement purposes. Exploratory factor analysis (EFA) was conducted on the data from the first quarter to identify the factor structure, followed by confirmatory factor analysis (CFA) on the rest of the data to confirm the EFA factor structure. A structural equation model (SEM) was fitted where the factors predicted overall satisfaction with the ED visit. Finally, measurement invariance was conducted to examine if patients and families perceived the factors related to ED services alike.

**Results:**

Two factors were found to be predictive of satisfaction: clinical team and system processes. The SEM showed that system process was a statistically significant predictor of overall satisfaction, while clinical team predicted overall satisfaction to a smaller extent. Multi-group CFA showed that the factor structure fitted neither family nor patient groups adequately. The instrument did not exhibit partial invariance.

**Conclusion:**

This study found that system process was the best predictor of overall satisfaction. Furthermore, this study showed that the same instrument might not reliably compare the perceptions of patients and families.

## Introduction

Patient satisfaction is a key metric used to assess service quality in emergency departments[1]. It describes how patients perceive and value their care. Patients’ satisfaction with their emergency care has been shown to affect their compliance with discharge instructions including medications usage as well as follow up visits [2, 3]. It has also been found to influence future ED choice as well as the likelihood of recommending the ED to others [3, 4]. Furthermore, improving patient-physician interaction has been shown to improve job satisfaction of healthcare professionals and ED staff, thus contributing to a positive work environment[3, 5].

Existing ED studies have identified several factors associated with improved patient satisfaction. These include high triage acuity level of the patient[3], strong interpersonal skills and empathy of the clinical staff[6] as well as the education patients received about their condition[7]. On the other hand, perceived and expected long waiting times negatively correlate with patient satisfaction [8–11].

While multiple studies have explored predictors of patient satisfaction, to our knowledge, no study has looked at family satisfaction with the care their loved ones receive in the ED. This is important because patients often lean on family for extra support during their ED visit, make them integral to the decision-making process and rely on them as carriers of information during the follow up phase[12]. In addition, no study has examined whether the items that measure patient and family members’ satisfaction function identically across the patient and family member groups. Without testing for measurement invariance, comparison of statistics across the groups are invalid[13, 14]. Measurement invariance is the property that is exhibited by instruments without measurement bias, that is, given the same score on an underlying construct, respondents from different groups would be expected to answer identically. Finally, understanding what the predictors of satisfaction are within the Lebanese cultural context are important and can build on the growing literature on contextualizing health care to cultural needs[15].

Additionally, only few studies have investigated perceptions of the ED services using multivariate analyses[5, 16]. Studying a fundamentally multivariate construct such as ED services using univariate analyses oversimplifies and ignores the inherent complexity of the construct. Multiple hypothesis testing in such situations can lead to increased experiment wise type-I error rate. The few that have used multivariate analyses, have relied on statistical significance testing (SST) rather than a modeling approach which is susceptible to all drawbacks of SST such as sensitivity to large sample sizes, reliance on an arbitrary cut-off p-value, and lack of replicable results (see [17, 18]).

To this end, the present study investigated the factors that best predicted overall satisfaction with the ED visit in a Lebanese cultural context. The study also examined whether factors that measure satisfaction with the ED are perceived identically between patients and their families.

## Methods

### Study design and setting

This study utilized a secondary analysis of de-identified data of survey responses collected by our Patient Affairs office from January 2016 to September 2016. The study setting is one of the largest EDs in Lebanon, seeing approximately 57,000 ED visits annually at a tertiary care academic medical center. The majority of ED patients (78%) are discharged home. As part of routine care, a Patient Affairs Office handles all patient complaints and administers patient satisfaction surveys on care received at our inpatient units, emergency department, and specialty ambulatory clinics. The Institutional Review Board at the American University of Beirut (AUB) approved this study.

### Survey instrument

In 2013, the ED leadership team and Patient Affairs developed a patient satisfaction survey based on existing ED surveys published in the peer-reviewed literature [5, 10, 19]. They customized these questions to the American University of Beirut Medical Center (AUBMC) setting based on patient complaints and process improvement initiatives within the ED. The survey included 30 questions that focus on five major domains including admission and discharge process, courtesy of staff, care and communication of clinical team, length of stay (LOS), and overall satisfaction (see S2 File).

Additionally, relevant patient demographic information was included.

### Data collection

The administration of the survey began in 2016 with the goal of surveying 2% of discharged ED patients). Patient Affairs received a daily list of all patients who presented to the ED. Patients discharged home from the ED were randomly selected by choosing every third patient and calling them within 2 days of discharge for survey participation. Families were only surveyed if the patient was less than 18 years of age or if the patient was not able to complete the survey for medical reasons and a family member who accompanied the patient throughout their ED visit was willing to complete it. A verbal consent was obtained prior to administering the questionnaire. In addition, all respondents were assured confidentiality of their responses. The staff members administering the phone surveys were trained in surveying techniques.

## Data analysis

The questions about patient satisfaction of the ED were rated by the respondents on a categorical scale with 1-very poor, 2-poor, 3-fair, 4-good, and 5-very good. Because the data were ordinally scaled, we used polychoric correlations for the analyses[20]. We screened the items for multicollinearity by examining their correlations. In item pairs that had correlations greater than 0.9, we deleted one of the items because essentially both items were providing the same information.

**Fig 1:**
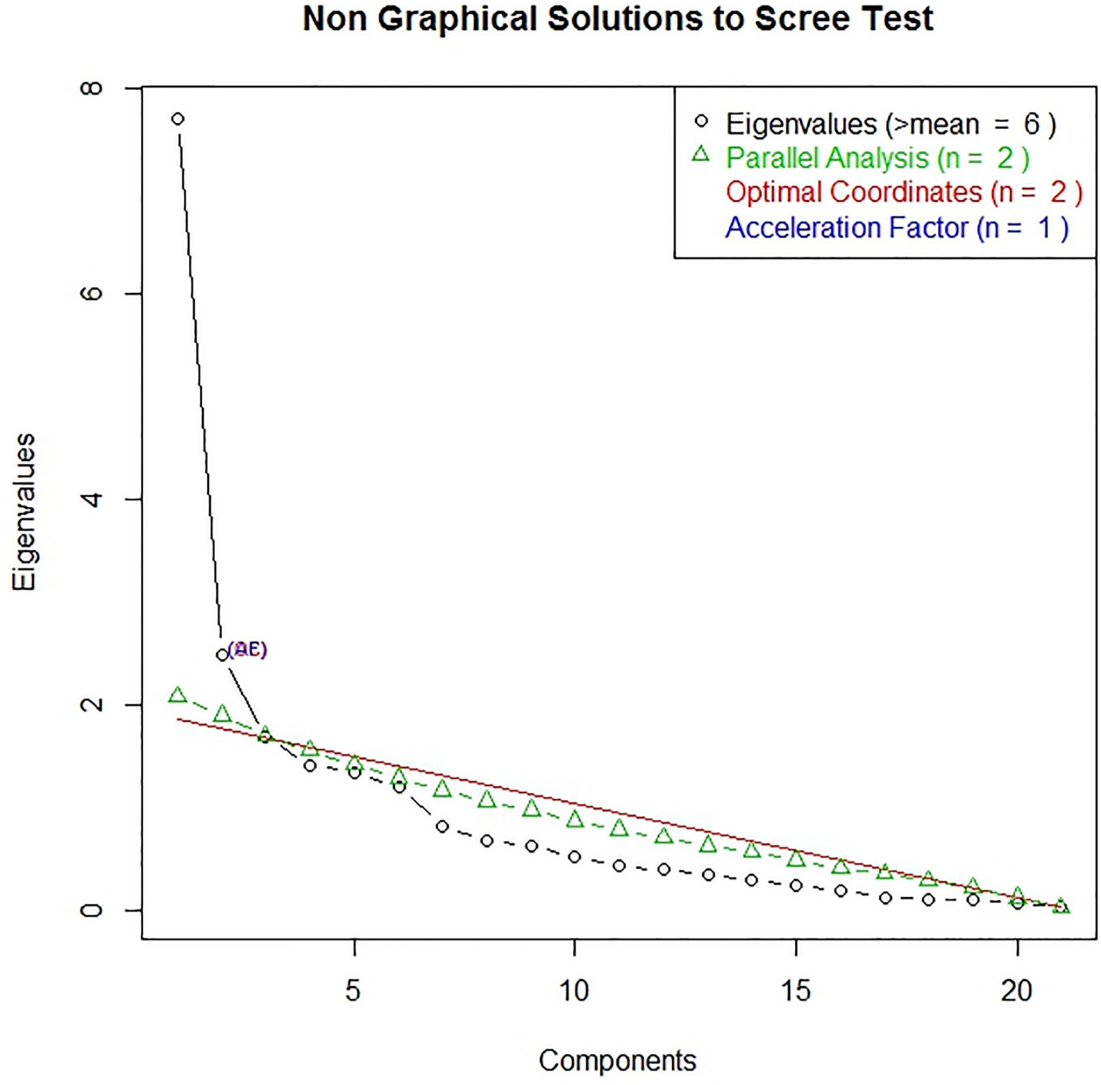
Non graphical solutions to scree test.

First, we conducted parallel analysis[21, 22] on the data from the first quarter to determine the number of factors to retain. Following this, we conducted an exploratory factor analysis (EFA) using principal axis factoring (PAF) on the first quarter and a confirmatory factor analysis (CFA) on the rest of the data. Instead of the five domains covered by the survey, parallel analysis indicated that two factors be retained as shown in Fig 1. EFA yielded two factors: system processes courtesy of staff, admissions and discharge process, the cleanliness of the ED, the respect of confidentiality and privacy, the overall noise of the ED, and LOS all indicated one factor)and clinical team (various aspects of care provided by the nurses and physicians). CFA on the second and third quarters based on the PAF was fitted to ensure that the factor structure held for the rest of the data as well. Model modification was done based on suggestions by the program to correlate errors of items but only if they made substantive sense. In order to predict how each factor predicted overall satisfaction of the ED, a SEM was fitted with the two factors predicting overall satisfaction with the ED and likelihood of recommending the ED to others.

Model fit was determined based on statistically non-significant χ^2^ value, CFI (> 0.95, Hu and Bentler, 1999), RMSEA and SRMR (< 0.08, Hu and Bentler, 1999), and RMSEA < 0.05 (good fit) and 0.05 < RMSEA < 0.08 (medium but acceptable fit, Browne & Cudeck, 1993). Finally, CFA and parallel analysis followed by PAF was conducted on patient and family data in order to test if the factor structure for the entire group held for each of these groups. For SEM that predicts overall satisfaction and measurement invariance, data from the entire sample of four quarters were used. Statistical software program R and package lavaan were used to conduct the statistical analyses.

## Results

### Demographics

Of the 1494 patients contacted, 705 agreed to complete the survey yielding a response rate of 50.6%. Demographics for the data are shown in Table 1. Sixty-six percent of the respondents were patients and the rest were family. Age and gender were only collected for the last three quarters of the administration. Twenty two percent of these patients were below 18 years of age. The median age of the patients was 30 with an interquartile range of 19 to 50. Fifty two percent of the respondents were female. For all these patients, their family was interviewed. For those who were above 18, only 17% interviewed were family. Mann-Whitney test of overall satisfaction with the ED showed statistical non-significance with p-values of 0.79 and 0.45 for gender and first visit, respectively. This indicates that overall satisfaction with the ED was not influenced by gender and first visit. The educational level of the respondents was not related to their overall satisfaction with the ED.

**Table 1:** Demographics of patients.

		All N = 705
**Age, Median (IQR), years** *, *n = 590*		30.0 (19–50)
	<18 years	130 (22.0)
	≥ 18 years	460 (78.0)
Sex*, *n = 590*	Females	310 (52.0)
	Males	280 (47.5)
**Education, *n = 704***	Graduate, PhD	306 (43.5)
	Undergraduate	205 (29.1)
	Technical	14 (2.0)
	Secondary	109 (15.5)
	Primary	60 (8.5)
	Illiterate	10 (1.4)
**Guarantor type, *n = 703***	Third party payer	619 (88.1)
	Self-payer	84 (11.9)
**First visit to the ED**^+^**, *n = 475***	Yes	66 (13.9)
	No	409 (86.1)
**Person interviewed, *n = 705***	Patient	466 (66.1)
	Family member	239 (33.9)

*Age and Gender were only collected for the last three quarter

^**+**^ Data from last quarter is missing

### Exploratory factor analysis

During item screening, *Explanations the registration staff gave you* and *Cooperation/helpfulness of the registration staff* were deleted because they were similar to *Courtesy of the staff in the registration area*. Similarly, *Explanations the cashiers gave you* and *Cooperation/helpfulness of the cashiers* were deleted because they were similar to *Courtesy of the cashiers*. These indicated that the respondents did not clearly distinguish between courtesy, explanations, and cooperation of the registration staff and the cashiers. Additionally, *Skill of nurse at inserting an Intravenous line (IVL)* was deleted because more than 80% of the responses were missing. This is expected because patients may not feel qualified enough to judge whether or not the nurse was skilled while inserting an IVL, families may not know how the patient felt while the nurse inserted an IVL, and not all patients get an IVL inserted during their ED visit.

Parallel analysis indicated that two factors be retained. PAF factor coefficients for the two factors–clinical team and system processes items are shown in Table 2. The factor pattern coefficients for items on the factors they were hypothesized to indicate were greater than 0.38. This means that the items were good indicators of the factors and provides supportive evidence of convergent validity. Cronbach’s alpha for system process, clinical team, and the two factors together were .9 [.89, .92], .8 [.77, .83], and .87 [.95, .89], respectively indicating good support for internal consistency reliability of the factors. The correlation between the factors was 0.62 indicating that the factors were correlated but were distinct from each other. This provides some evidence of discriminant validity.

**Table 2:** Factor/pattern coefficients from exploratory factor analysis (EFA) and confirmatory factor analysis (CFA).

Items	Factor/pattern coefficients							
	EFA		CFA		Patient		Family	
	Cli	Sys	Cli	Sys	Cli	Sys	Cli	Sys
Q2- Courtesy of registration staff		0.81		0.70		0.66	0.23	0.44
Q5- Waiting time at registration area		0.68		0.55	-0.30	0.86	0.32	0.23
Q6- LOS before being seen by triage nurse	0.44	0.30		0.63		0.57	0.32	0.35
Q7- Nurses introduced themselves		0.69		0.62		0.64		0.39
Q8- Courtesy of nurses	0.21	0.64		0.77		0.70	0.24	0.52
Q10- Concern the nurse showed for your questions and worries	0.55	0.34	0.81		0.31	0.56		0.76
Q11- Communication of progress and delays by nursing staff	0.46	0.30	0.71		0.19	0.56		0.82
Q12- LOS before being seen by the ED physician	0.38	0.38	0.64			0.69	0.24	0.46
Q13- Physicians introduced themselves	0.46	0.32	0.73		0.33	0.46	0.58	0.20
Q14- Courtesy of the attending physician	0.70		0.83		0.56	0.36	0.90	
Q15- Explanations physician gave about your condition	0.95		0.86		0.91		0.85	
Q16- Communication of plan of care by physician	0.96		0.87		0.89		0.94	
Q17- Concern the physician showed for your questions and worries	0.70	0.19	0.88		0.78		0.75	0.16
Q18- Instructions the physician gave you about follow-up	0.79		0.76		0.81		0.61	0.17
Q19- Amount of time physician spent with you	0.61	0.21	0.79		0.73		0.65	0.17
Q20- Courtesy of the cashiers		0.62		0.74	0.17	0.59	0.53	0.16
Q23- Waiting time at the cashier		0.65		0.66		0.64	0.43	0.16
Q25- Overall cleanliness of the ED		0.61		0.57	0.16	0.53		0.54
Q26- Overall noise	0.07	0.63		0.60		0.63		0.64
Q27- Respect of confidentiality and privacy	0.16	0.50		0.65	0.17	0.52	0.21	0.39
Q29- Likelihood of recommending our ED to others	0.22	0.45		0.68		0.61		0.72

Coefficients less than 0.15 are not shown; Cli–Clinical team; Sys–System Processes

### Confirmatory factor analysis

A CFA model (CFA-a) was fitted to a dataset for the rest of the data. This model had adequate fit except for SRMR (Table 3). However, the model modification suggested that there were error correlations between the following item pairs:

1. Overall ED (LOS)–LOS before being seen by the ED physician;
2. Nurses introduced themselves–Physicians introduced themselves;
3. Waiting time at the registration area–Waiting time at the cashier
4. Waiting time at the registration area–LOS before being seen by the ED physician.

**Table 3:** Model Fit Indices for the confirmatory factor analysis (CFA) and the structural equation model (SEM).

Model*χ*^2^	*χ*^2^	df	pvalue	CFI	RMSEA [90% CI]	RMSEA.p-value	SRMR
CFA-a	421.51	188	<.01	0.96	0.06	0.11	0.08
					[0.05 0.06]		
CFA-b	357.32	184	<.01	0.97	0.04	0.89	0.08
					[0.04 0.05]		
SEM	497.22	222	<.01	0.97	0.05	0.55	0.07
					[0.04 0.06]		

It is logical that these items have correlations that can be explained by factors other than the two modeled; this is because of similarity in wording and explanations other than the underlying construct such as time and introduction by the clinical care professional. Therefore, a modified CFA model (CFA-b) was fitted to the data with errors from these items correlated. We retained this model because this model had slightly better fit than the previous model. Statistical non-significance of these models was not considered in model retention decisions because in reality, it is not always possible to obtain statistical non-significance for complex models such as those with more than 4 items per factor[23].

**Fig 2:**
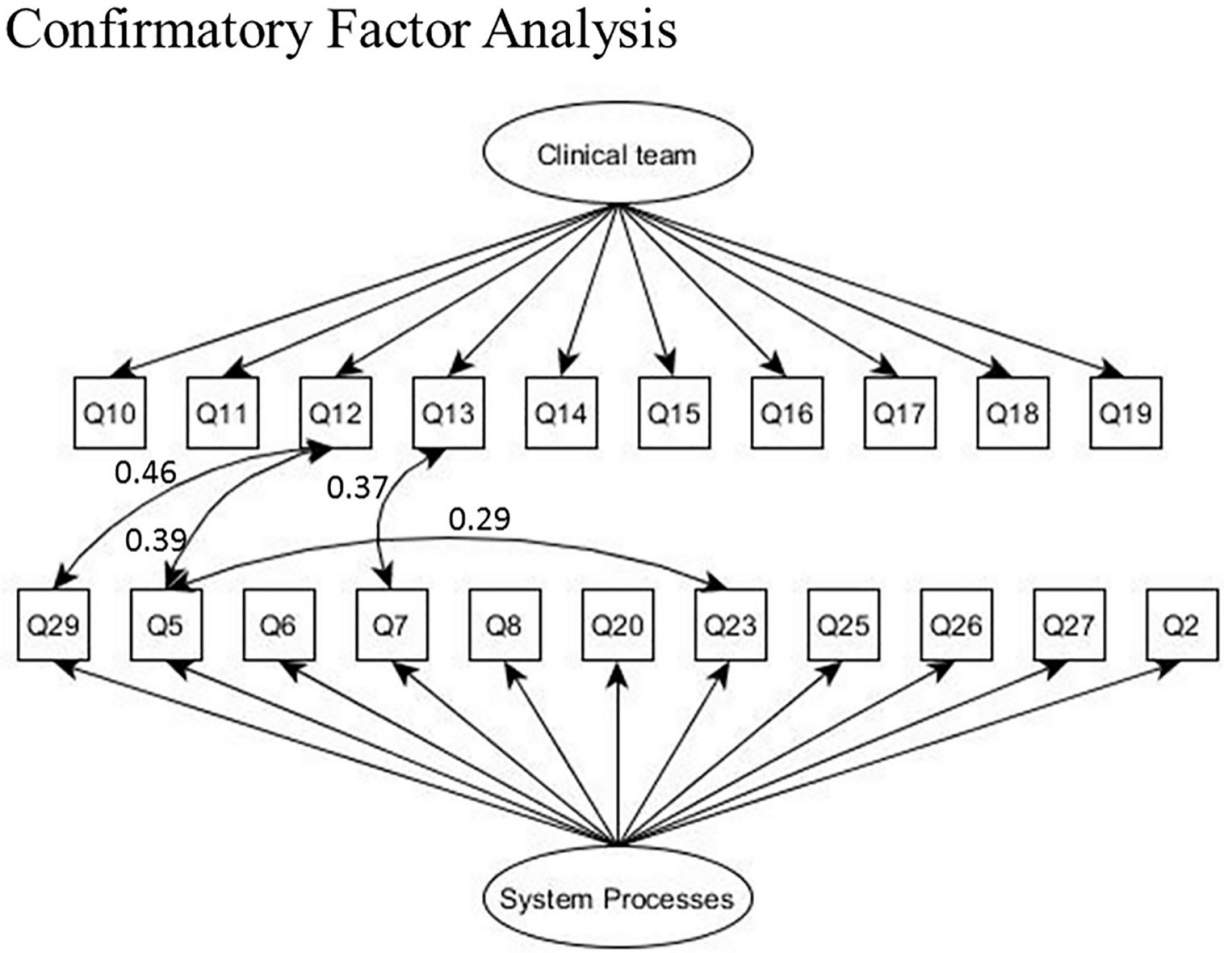
Confirmatory factor analysis.

**Fig 3:**
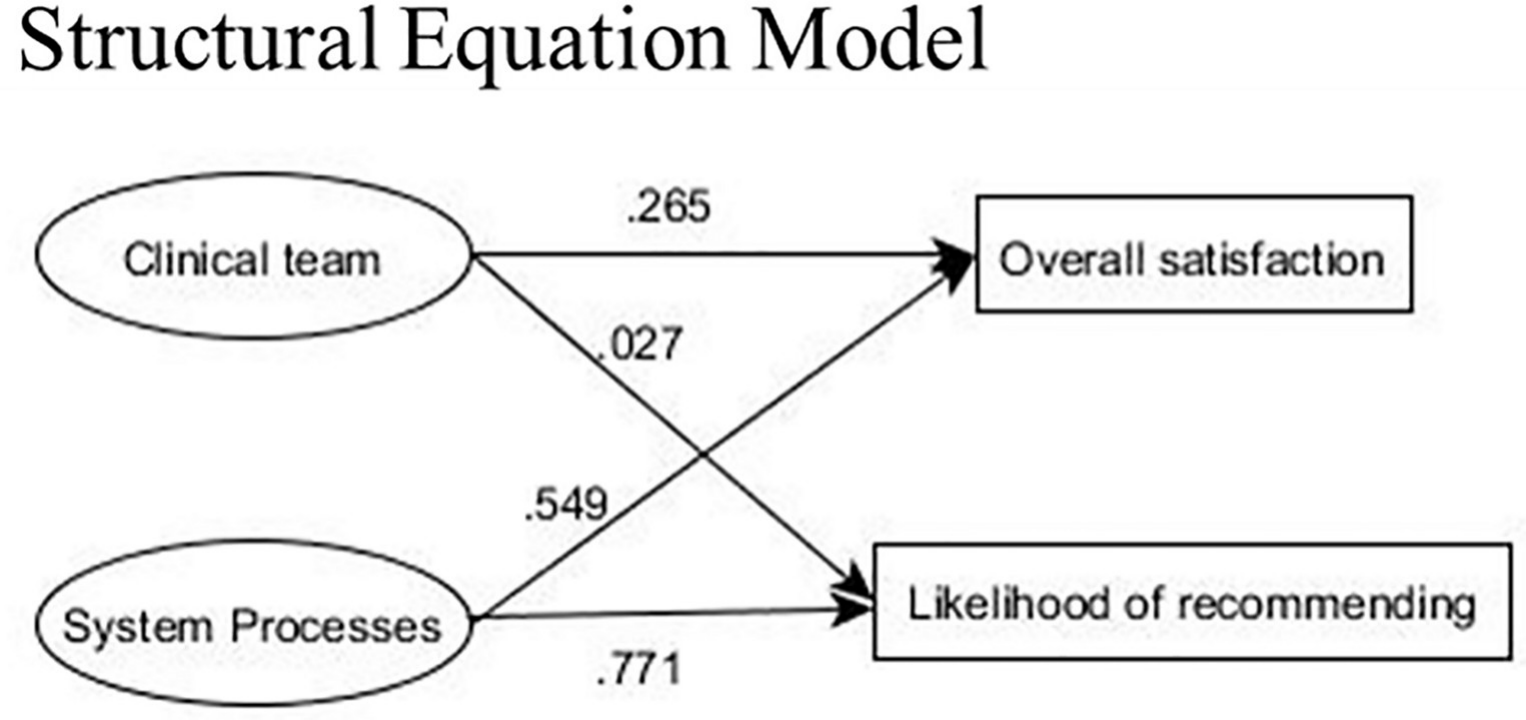
Structural equation model.

The factor pattern coefficients and the figure for CFA-b are shown in Table 2 and Fig 2, respectively. The factor correlation between clinical team and system process was 0.84. Next, the two factors, clinical team and system processes were regressed to predict overall satisfaction with the ED and the likelihood of recommending the ED to others as shown by the structural equation model (SEM) in Fig 3. Both system process (0.549, SE = 0.08) and clinical team (0.265, SE = 0.08) were statistically significant predictors of overall satisfaction. However, only system process (0.771, SE = 0.08) was a substantially large and statistically significant predictor of the likelihood of recommending the ED to others.

Our initial goal was to conduct measurement invariance to identify differences in patient and family perceptions of ED satisfaction. For this, the same factor structure and various parameters should be identical across groups. However, the simplest multigroup CFA model, that is, one with identical factor structure but varying parameters for both groups did not produce adequate fit. Therefore, we conducted PAF for both datasets individually.

Parallel analysis showed that 2 factors needed to be retained for both datasets. Therefore, PAF was conducted for family and patient datasets specifying retention of 2 factors to each dataset. The factor pattern coefficients revealed that the two groups define clinical team and system process differently (Table 2). Families perceive discharge as part of the clinical team while patients perceive discharge as part of the system process. In fact, family members perceive that admissions are part of the clinical team as seen by the small but not negligible factor pattern coefficients of these items on the clinical team factor (0.23–0.32). The fit indices of the configural invariance model that tests if the same factor structure holds true for both groups together did not pass the thresholds when fitted using multi-group CFA. Therefore, even partial invariance could not be established for the two groups.

Overall, these results suggest that the two groups define their perceptions of their satisfaction of ED services differently. Therefore, their perceptions cannot be compared directly. Neither can family’s satisfaction with the ED be used in place of patient’s satisfaction with the ED.

## Discussion

This study is the first to explore multivariate predictors of satisfaction with ED visit for patients compared to their accompanying family members. The results suggest that patients and families experience ED visits differently and that different aspects of the care predict their overall satisfaction with the visit. In cultural contexts where families are a key stakeholder in the care, understanding these differences is important for managers and for driving improvement efforts.

Studies have shown that waiting time [9], courtesy of the staff, physicians’ services [10], and cleanliness to be the strongest predictors of ED satisfaction. Our findings are in line with the literature. System processes were the best predictors of overall satisfaction with ED visit in our study, followed by clinical team. LOS was not directly regressed on overall satisfaction unlike in other studies because we fitted latent variable models as opposed to conducting univariate analyses, which are only concerned with single measured variables rather than constructs. Furthermore, system processes were better predictors of likelihood of recommending the ED to others than satisfaction with the clinical team.

Several studies have revealed that waiting times [10] in the ED is the largest predictor of patient satisfaction. Our study shows that waiting time alone is not a major predictor of patient satisfaction, but the complex factor called system processes, which also includes the admission process, discharge process, cleanliness, and overall noise in the ED is.

Margaret et al. (2002) concluded that factors influencing the overall satisfaction were similar amongst both children and parents; patient satisfaction was associated with the adequacy of information provided, shorter waiting room times, and the quality of provider-patient interactions. While our study did not compare the family member’s perception directly with the patient who they accompanied (i.e. paired samples), it did include surveys of family members who had accompanied both adult and pediatric patients (i.e. independent samples). Contrary to Margaret et al.’s study, our results demonstrated that the perceptions of family members about their visit to the ED could not be compared to those of patients. While patients see only the aspects of physicians as indicators of clinical team care, families associate physician-related questions as well as questions related to the discharge process as indicators of clinical team care. This could be related to the existing process in our ED where the clinical team directs patients to the financial clearance process at discharge, in addition to being the main driver of patient discharge including patient education, and follow-up instructions. This highlights the need to handle family members’ perceptions separately and the inability to use their experiences of the ED as a proxy for patients’ perceptions. In sum, even simple comparisons of average scores by patients and families are not valid.

## Limitations

The study was limited to one hospital in the Lebanese health system and may not be generalizable to other hospitals and other healthcare systems. In addition, our study did not compare the family’s perception with the perception of the patients they accompanied; rather it compared patients and families of other patients. Furthermore, the majority of respondents were patients and only 44% were family members thus limiting representation of the latter group. Finally, our sample size restricted us to fit only simple models.

## Conclusion

Even though patients visit the hospital expecting high quality clinical service, their overall satisfaction is affected by other system aspects of clinical care indicated by the admission process, the discharge process, the overall LOS, the cleanliness of the ED, the respect of confidentiality and privacy, and the overall noise of the ED, rather than actual clinical care itself. Family’s experience of ED care is not the same as the patient’s and survey instruments need separate validity testing to be used for this group in settings where families are key stakeholders to care. Understanding the drivers of both patients’ and families’ satisfaction is an area of needed research. Future research may also focus on establishing a meaningful cut-point on the factor scores to determine if the care was satisfactory.

## Supporting information

S1 File De-identified data of survey responses collected between January 2016 and September 2016. (CSV)

S2 File English survey instrument. (PDF)

S3 File Arabic survey instrument. (PDF)
