# Supplementary material for: Comparing patients and families perceptions of satisfaction and predictors of overall satisfaction in the emergency department
Source: PLoS One. 2019 Aug 13;14(8):e0221087. doi: 10.1371/journal.pone.0221087 (PMC6692004; doi:10.1371/journal.pone.0221087)
Supplement: S2 File — (PDF) [file pone.0221087.s002.pdf]

AMERICAN UNIVERSITY of BEIRUT MEDICAL CENTER
المركز الطبي في الجامعة الأميركية في بيروت

DEPARTMENT OF EMERGENCY MEDICINE
PATIENTS SATISFACTION SURVEY

Date: ------/------/------
Age:
Gender: ☐ F ☐ M

ED disposition: ☐ Discharged Home ☐ Admitted
Guarantor Type: ☐ Insurance ☐ Self payer ☐ HIP ☐ NSSF ☐ Others
Level of education: ☐ Graduate/PHD ☐Undergraduate ☐ Technical ☐ Secondary (BaccII) ☐ Primary (Brevet) ☐ Illiterate
Time of visit: ☐ Morning ☐ Evening ☐ Night
Living location: ☐ Urban ☐ Rural
Patient's first visit to ED: ☐ Yes ☐ No
Who has completed the questionnaire: ☐ Patient ☐ Family member ☐ Other

Why did you chose the Emergency Department at AUBMC?

A. ☐ I chose AUBMC ED because it is conveniently located or close to my residency / Distance to the hospital
B. ☐ I chose AUBMC ED because I trust AUBMC
C. ☐ I chose AUBMC ED because trust the ED at AUBMC and the ED provider team
D. ☐ I chose AUBMC because my primary care provider or specialist is at AUBMC
E. ☐ I chose AUBMC ED because I had a previous experience in the ED that was good.
F. ☐ I chose AUBMC ED because of the recommendations of trusted personal sources (physician referrals, friends and relatives)
G. ☐ Other

Determine your satisfaction level in regard to each of the below items

1	2	3	4	5	NO
**Very Poor**	**Poor**	**Fair**	**Good**	**Very good**	**Not Observed**

	1	2	3	4	5	NO
A. Admission						
Cooperation/helpfulness of the registration staff						
Waiting time at the registration area						
B. Nursing						
LOS before being seen by triage nurse						
Nurses introduced themselves						
Courtesy of nurses						
Skill of nurse at inserting an IV						
Concern the nurse showed for your question and worries						
Communication of progress and delays by nursing staff						
C. Medical Team						
LOS before being seen by the ED physician team						
Physicians introduced themselves						
Courtesy of the attending physician						
Explanations the physician gave you about your condition						
Communication of plan of care by physician						
Concern the physician showed for your questions or worries						
Instructions the physician gave you about follow-up care						
Amount of time the physician spent with you						
D. Discharge						
Courtesy of the cashiers						
Explanations the cashiers gave you						
Cooperation/helpfulness of cashiers						
Waiting time at the cashier's station for payment						
E. Overall satisfaction						
Overall satisfaction with your visit to the ED						
Overall cleanliness of the ED						
Respect of confidentiality and privacy						
Likelihood of your recommending our ED to others						
Overall ED LOS						

Do you have any suggestions to improve the service at the AUBMC ED?

____

____
