# Supplementary material for: Comparing patients and families perceptions of satisfaction and predictors of overall satisfaction in the emergency department
Source: PLoS One. 2019 Aug 13;14(8):e0221087. doi: 10.1371/journal.pone.0221087 (PMC6692004; doi:10.1371/journal.pone.0221087)
Supplement: S3 File — (PDF) [file pone.0221087.s003.pdf]

المركز الطبي في الجامعة الأميركية في بيروت

قسم الطوارئ

استطلاع رأي المرضى

موافقة شفاهية:

أنا المدعو_______________ اتصل بك من إدارة المركز الطبي في الجامعة الأميركية في بيروت لكي أسألك عن اقامتك في قسم الطوارئ.

هذه الدراسة تقام لتحسين نوعية الخدمة الذي يقدمها المركز الطبي للمرضى. رأيك جوهري ويهمنا كثيراً. إشتراكك في هذه الدراسة هو إختياري.

المقابلة سوف تأخذ 10 دقائق من وقتك والمعلومات التي ستقدمها مهمة لنا لكي نستمر في تطوير وتحسين نوعية الخدمة لمرضانا. أجوبتك ستبقى سرية واسمك أيضاً، ولن يستطيع أحد أن يصلها بك أو الوصول إليها.

لديك الحق في عدم المشاركة في هذه الدراسة وهذا لن يؤثر على نوعية الخدمة المقدمة لك في المركز الطبي في المستقبل، ولكن إذا اخترت المشاركة، رأيك سوف يلعب دوراً هاماً في تحسين نوعية الخدمة في قسم الطوارئ في المركز الطبي في الجامعة الأمريكية في بيروت.

هل تود المشاركة:

- □ غير موافق
- □ موافق

التاريخ: ------/------/----

العمر: ____

الجنس:

- □ ذكر
- □ أنثى

نوع الكفيل:

- □ تأمين
- □ صندوق وطني للضمان الإجتماعي
- □ على حسابه
- □ جهة أخرى
- □ HIP

المركز الطبي في الجامعة الأميركية في بيروت

قسم الطوارئ

استطلاع رأي المرضى

مستوى التعليمي:

- ☐ دراسات عليا/دكتورا
- ☐ جامعي
- ☐ مهني
- ☐ ثانوي (بكالوريا قسم ثاني)
- ☐ متوسط (بريفيه)
- ☐ أمي

وقت الزيارة:

- ☐ صباحاً
- ☐ مساءً
- ☐ ليلاً

محل الإقامة:__________

زيارة المريض الأولى في قسم الطوارئ؟

- ☐ نعم
- ☐ لا

من قام بملء الإستطلاع؟

- ☐ المريض
- ☐ فرد من العائلة
- ☐ شخص آخر

لماذا اخترت قسم الطوارئ في المركز الطبي في الجامعة الأمريكية في بيروت؟

- ☐ لموقعه الملائم من موقع سكني/ بسبب المسافة القريبة إلى المركز الطبي
- ☐ لأني أثق بهذا المركز
- ☐ لأني أثق بقسم الطوارئ في المركز وبالفريق العامل في هذا القسم
- ☐ لأن الطبيب الذي يهتم بي أو طبيب الإختصاصي متواجد في هذا المركز
- ☐ سبق وكانت لي تجربة ناجحة في هذا القسم
- ☐ بسبب توصيات من أشخاص موثوقين (الطبيب المعالج، أصدقاء، أقارب)
- ☐ لأسباب أخرى: ______________________

<u>حدد مستوى رضاك عن كل من مسائل المذكورة أدناه:</u>

1	2	3	4	5	1-
**سيئ جداً**	**سيئ**	**محايد**	**جيد**	**جيد جداً**	**غير مطابق**

المركز الطبي في الجامعة الأميركية في بيروت

قسم الطوارئ

استطلاع رأي المرضى

**أ: عملية الدخول**	**1**	**2**	**3**	**4**	**5**	**1-**
لباقة فريق العمل عند الدخول						
تفسيرات التي أعطاها موظفي الدخول عن الأوراق المطلوبة						
تعاون موظفي الدخول						
مدة الإنتظار عند مكتب الدخول						
**ب: فريق التمريض**	**1**	**2**	**3**	**4**	**5**	**1-**
مدة الإنتظار قبل قدوم الممرض/الممرضة						
قام الممرضون/الممرضات بالتعريف عن أنفسهم						
لباقة فريق التمريض						
ابلاغك بالتطورات أو التأخير من قبل فريق التمريض						
إصغاء فريق التمريض لاسئلتك ومخاوفك						
التفسيرات التي أبلغك بها فريق التمريض (مثل: فحوصات/عمليات)						
تلبية فريق التمريض لطلباتكم واحتياجاتكم						
تقييم الألم من قبل فريق التمريض (من ناحية المدة الزمنية، الموقع، الشدة)						
**ت: فريق الطبي**	**1**	**2**	**3**	**4**	**5**	**1-**
مدة الإنتظار قبل قدوم فريق عمل طبيب الطوارئ						
قام الأطباء بالتعريف عن أنفسهم						
لباقة الطبيب المعالج						
التفسيرات التي أبلغك بها الطبيب عن حالتك الصحية						
ابلاغك من قبل الطبيب عن الخطة العلاجية التي سيتم اتباعها						
الإهتمام الذي أظهره الطبيب لأسئلتك ومخاوفك						
التعليمات التي أعطاها الطبيب عن متابعتك لحالتك الصحية						
الفترة الزمنية التي امضاها الطبيب معك						
**ث: عملية الخروج**	**1**	**2**	**3**	**4**	**5**	**1-**
لباقة موظفي الصندوق						
التفسيرات التي أبلغك بها موظفي الصندوق						
تعاون موظفي الصندوق						
مدة الإنتظار عند الصندوق للمحاسبة						
**ج: مستوى الرضا**	**1**	**2**	**3**	**4**	**5**	**1-**
رضاك بشكل عام عن زيارتك إلى قسم الطوارئ						
النظافة العامة في قسم الطوارئ						
مستوى الضجيج في قسم الطوارئ						

المركز الطبي في الجامعة الأميركية في بيروت

قسم الطوارئ

استطلاع رأي المرضى

						إحترام السرية والخصوصية
						إحتمال توصيتك بزيارة قسم الطوارئ لدينا إلى أشخاص آخرين
						مدة البقاء في قسم الطوارئ بشكل عام

هل لديك أية إقتراحات من شأنها تحسين الخدمة في قسم الطوارئ في المركز الطبي في الجامعة الامريكية في بيروت؟

____

____

____
